# Hashimoto’s thyroiditis: from pathogenesis to clinical management

**DOI:** 10.3389/fendo.2026.1729316

**Published:** 2026-02-02

**Authors:** Linghui Wang, Xi Zhu, Shuting Xu, Bin Zhou, Yong Wu, Zhouting Li, Yanjie Zhao, Shuhui Li, Feng Cheng, Lei Zhu

**Affiliations:** 1 Graduate Joint Training Base of Zhejiang Chinese Medical University (Lishui Joint Training Base - Lishui Municipal Central Hospital), Hangzhou, Zhejiang, China; 2 Department of Head and Neck Surgery, The Fifth Hospital Affiliated to Wenzhou Medical University, Lishui Central Hospital, Lishui, Zhejiang, China; 3 Hangzhou Medical College, Hangzhou, Zhejiang, China

**Keywords:** autoimmunity, Hashimoto’s thyroiditis, hypothyroidism, immunomodulation, thyroglobulin antibody, thyroid peroxidase antibody

## Abstract

Hashimoto’s thyroiditis (HT) is a chronic autoimmune thyroiditis characterized by thyroid-specific autoantibodies (TPOAb, TGAb) positivity and lymphocytic infiltration, and is a major cause of hypothyroidism in iodine-sufficient regions. Epidemiological data show a significant increase in the prevalence of HT, which is about four times more common in adult women than in men. The pathogenesis of HT involves a complex interaction of genetic susceptibility, environmental factors, and immune regulation. Its clinical diagnosis is mainly based on serological tests (TPOAb/TGAb) and thyroid ultrasound features. The current treatment of Hashimoto’s is based on levothyroxine (T4) replacement. This article presents a narrative review of the pathogenesis, status, and challenges of treatment of HT to provide a theoretical basis for optimizing clinical practice and basic research.

## Introduction

Hashimoto’s thyroiditis (HT) is characterized by thyroid peroxidase antibody(TPOAb) and thyroglobulin antibody (TGAb) positivity, lymphocytic infiltration, and progressive hypothyroidism (1–3); it is one of the most common organ-specific autoimmune diseases (4). In recent years, the prevalence of HT has increased significantly worldwide, especially in iodine-sufficient areas, and the incidence of HT in adult females can be 4–10 times higher than that in males, making it an increasingly serious public health problem (5). The pathogenesis of HT is characterized by the triple interaction of genetics-environment-immunity (1). The complex pathogenesis of HT, which results in a highly heterogeneous clinical presentation, with the clinical symptoms ranging from positive subclinical antibodies to significant hypothyroidism, spans several decades, making early diagnosis and intervention very difficult.

Currently, three major bottlenecks still exist in clinical diagnosis and treatment: first, the traditional diagnostic model relying on antibody detection and ultrasound has a limited ability to predict the progression of the disease; secondly, levothyroxine(LT4) replacement therapy can correct hypothyroidism but cannot block the autoimmune process; and thirdly, there is a lack of consensus on the intervention strategy for patients with subclinical stage. With the application of single-cell sequencing, artificial intelligence, and other technologies, the study of HT is moving towards a new stage of molecular typing and precision medicine. In this article, we review the pathogenesis, current status of diagnosis and treatment of HT, and other related research progress, aiming to provide new perspectives for optimizing clinical practice and translational research.

## Background and epidemiology

Hashimoto’s thyroiditis is an autoimmune disease characterized by the persistent presence of specific autoantibodies (thyroid peroxidase antibodies and thyroglobulin antibodies) as well as lymphocytic infiltration, also known as autoimmune or chronic lymphocytic thyroiditis (1, 6, 7). In iodine-deficient regions, HT is the main cause of hypothyroidism, and Hashimoto’s is also characterized by hypothyroidism (2, 3). The incidence of HT is on the rise globally, with a prevalence of only 2% a decade or so ago, but today the prevalence of the disease is 5-10% (8, 9). Adult females have approximately four times the risk of HT compared to adult males. And according to a study in the UK, the age at which autoimmune diseases, including HT are diagnosed is concentrated at around 54 years of age (5). In addition, HT is a potential risk factor for thyroid cancer (10). The increasing incidence of HT has necessitated a more in-depth understanding of the disease to diagnose and intervene in its development accurately.

## Etiology and pathogenesis

Hashimoto’s thyroiditis is an organ-specific autoimmune disease (4), and its pathogenesis involves the interaction of environmental, genetic, and immunological factors (1). In recent years, more and more studies have shown that the core pathological process of the disease is the abnormal recognition and attack of thyroid antigens by the immune system, leading to an imbalance of immune homeostasis and the development of autoimmune diseases (11). For example, autoreactive T cells trigger a humoral response in some susceptible individuals, generating antibodies against thyroid antigens (12), which in turn destroy the follicular cells of the thyroid and cause them to become hypothyroid; Th1 cells in the Th1/Th2 subset of helper T cells mediate the cellular immune response through activation of delayed-acting hypersensitivity, while Th2 regulates antibody production and humoral immune response, resulting in a cellular imbalance that leads to apoptosis of thyroid follicular cells (13). In addition, certain strains of intestinal flora, such as Lactobacillus, can increase the number of Th17 cells capable of expressing pro-inflammatory factors to alter the ratio of Th17 cells to Tregs, thereby disrupting immune homeostasis; a meta-analysis revealed that patients with Hashimoto’s thyroiditis have a higher gut microbiota richness index compared to healthy controls, while beneficial bacterial species such as Bifidobacterium are relatively reduced (14–16). Thus, this evidence implicates gut microbiota as a contributing factor in Hashimoto’s thyroiditis.

In the 1970s, the HLA gene was first found to be associated with HT (17), and as genetic techniques continue to evolve, the understanding of the disease has expanded from single-gene correlations to multi-gene interactions. Currently, genetic factors are broadly classified into three categories, namely human major histocompatibility complex genes, immunomodulatory genes, and thyroid-specific genes (18). As early as the 1990s, it was established that HLA-DR in the human major histocompatibility complex (MHC) class II genes is associated with susceptibility to the disease and also has a strong genetic association with the disease (19, 20). HLA is the most common gene associated with the onset of HT, with HLA-DR3 being the main risk haplotype in Caucasians and being more pronounced in the over-50-year-old female population, while HLA-DR5 is significantly associated with Asian populations (14, 21–23).In addition, NFE2L2 and SELENOS alleles, cytotoxic T-lymphocyte antigen 4(CTLA-4), FOXP3, and intracellular tyrosine phosphatase (PTPN22) have been associated with the risk of Hashimoto’s (24–27). Genetic variants enhance the binding of self-peptides to MHC by altering the process of autoantigen presentation and amplify the stimulation of T-cells, which in turn affects the normal function of thyroid cells (18). Furthermore, genetic variations in thyroid-specific genes-including the thyrotropin receptor (TSHR), thyroglobulin gene (TG), and thyroid peroxidase gene (TPO), play a key role in the development of the disease (28). These genes encode thyroid functions and can exacerbate thyroid follicular destruction in HT patients by altering the structure and expression levels of the corresponding antigens, thereby driving the development of HT (29). The rs4411444 GG genotype and the rs4903961 C allele in the TSHR gene enhancement region are most closely associated with this disease (30). Also, thyroid-specific genes are expressed in the central nervous system, allowing the development of Hashimoto encephalopathy (31). In addition to the above genes, recent studies have found that genes such as those encoding the vitamin D receptor, those involved in selenium metabolism, and those related to microbial susceptibility are also associated with the development of Hashimoto’s (32–35).

Although immune and genetic factors are predominant in the pathogenesis of HT, the triggering role of environmental factors is also indispensable. Among these factors, environmental influences such as excessive iodine intake, viral infections, and vitamin D deficiency contribute to the thyroid autoimmune response by disrupting immune tolerance and synergizing with genetic predispositions to cause the disease. Iodine is a component of thyroid hormones, thyroxine (T4) and triiodothyronine (T3), and is a key player in thyroid hormone synthesis in thyroid follicular cells (36); however, excessive iodine intake also increases the risk of autoimmune thyroid disease (37). Viral infections cause HT by triggering an immune response in the host that activates autoreactive T cells (38). Vitamin D plays a role in the autoimmune process and thus in the development of autoimmune diseases (39).

In conclusion, the pathogenesis of HT is the result of a dynamic interaction between immune intolerance, genetic susceptibility, and environmental triggers. Genetic variants provide the basis for the autoimmune response, while environmental factors ultimately lead to progressive destruction of thyroid follicular cells by breaching the threshold of immune tolerance.

## Clinical presentation and diagnosis

Hashimoto’s thyroiditis is a chronic inflammatory disease characterized by an autoimmune attack specific to the thyroid, with a highly variable clinical presentation ranging from an asymptomatic subclinical state to significant hypothyroidism. As the disease advances, lymphocytic infiltration of the thyroid tissue and destruction of follicular cells can cause the thyroid to enlarge or atrophy, ultimately leading to abnormal thyroid function (40). Because early symptoms are often vague and nonspecific, clinical diagnosis frequently depends on serological markers [e.g., antithyroid peroxidase antibody (TPOAb), antithyroglobulin antibody (TGAb)], imaging, and evaluation of thyroid function. Symptoms of HT can be local or systemic. Local symptoms mainly result from compression caused by an enlarged thyroid gland, such as tracheal compression. Systemic symptoms are primarily due to hypothyroidism, as the hormones produced by the thyroid are insufficient to meet the needs of peripheral tissues (6, 41).

The diagnosis of the disease is mainly dependent on clinical symptoms and the presence of TPOAb, which is positive in about 95% of Hashimoto patients (6). When TPOAb and TGAb are overexpressed, IgG4 is informative for diagnosis and typing assessment (29). Whereas in TPOAb-negative patients, ultrasound plays an important role, the ultrasound features of HT patients include glandular echogenicity, inhomogeneity, abundant blood flow, and the presence of small cysts (40). Ultrasound is also used to differentiate HT from other thyroid diseases (42). Due to the low resolution and relatively more extraneous noise of conventional ultrasound, the feature-level enhanced integrated network (FBENet) was introduced to solve these problems with an average accuracy of 82.92%. FBENet is a deep learning model designed to accurately distinguish Hashimoto’s thyroiditis from normal thyroid tissue in ultrasound images (43). In conclusion, for the diagnosis of HT, the combination of ultrasound and thyroid antibody test results is more informative (44, 45).

## Treatment and management

The key to the current treatment of HT is the regulation of thyroid hormones as a means of improving thyroid function and thereby slowing the progression of the disease. When the patient does not have thyroid function changes, it is often sufficient to follow the patient with follow-up visits to monitor changes in thyroid function. When hypothyroidism occurs, hormone replacement is often achieved with oral Levo-thyroxine 4 (LT4) tablets, which need to be taken for life (40). LT4 can partially reverse HT-induced muscular, renal, and lipid dysfunctions and significantly reduce the symptoms associated with HT in patients (46). However, even with oral LT4 hormone replacement therapy, some clinical symptoms persist, such as fatigue, arthralgia, dry eyes, dry mouth, etc (47). Therefore, LT4 and T3 combination therapy has been proposed. Combined with fourteen published studies showed that the combination did not show significant benefits (48), so this option is still controversial (40). In addition to Western drug therapy, traditional Chinese medicine (TCM) also plays an important role in the treatment of this disease. More and more studies have shown that certain Chinese medicines have better efficacy in lowering the level of thyroid antibodies and improving clinical symptoms, such as Chai Xiang Dispersing Knot Granules, Xia Kou Dispersing Gall Soup, Dispersing Knot Soup for Galls, and Xiaoyao-san (49, 50); according to a randomized controlled study, Xiaoyao-san preparation were able to significantly lower the level of TPOAb, and reduce antibody levels (when combined with LT4, it could even more closely reduce antibody levels (51). In addition, some studies have shown that traditional Chinese medicine acupoints can improve the clinical symptoms of patients with this disease and can also reduce the level of serum antibodies, thus slowing down the process of clinical hypothyroidism in patients (52). In addition to pharmacological treatment, studies have also shown that total thyroidectomy is decisive for improving the relief of autoimmune features such as vitiligo, pernicious anemia, thrombocytopenia, etc., exhibited by HT (53), but at present, HT exhibits a variety of immune-related features that have not been included in the indications for surgery.

Selenium supplementation is also currently important in the treatment of clinical HT as an element that plays a crucial role in the secretion of thyroid hormones. Selenium supplements can restore thyroid function to normal in some patients with subclinical hypothyroidism (54). The addition of selenium to the treatment regimen for Hashimoto’s thyroiditis, as shown in a prospective randomized controlled study, led to a significant reduction in TPOAb and TGAb levels in these patients (33). Since vitamin D is involved in the development of the disease, vitamin D supplementation has also been shown to be important in the treatment of thyroid dysfunction (32). Although it does not improve the function of the thyroid gland itself, it can change the titer of TPOAb (55),a prospective, randomized, controlled clinical study demonstrated that after 6 months of vitamin D supplementation therapy (both vitamin D alone and vitamin D in combination with LT4) resulted in thyroid peroxidase antibody compared to untreated controls a significant reduction in titre levels (56). In addition, lifestyle interventions play a pivotal role in the treatment of the disease. For example, excessive iodine intake can induce thyroid autoimmunity by increasing the immunogenicity of Tg in genetically susceptible individuals, and HT patients should avoid a high iodine diet (57). Furthermore, many researchers have suggested that other diets are also beneficial for patients with the disease, e.g., low-carbohydrate diets, gluten-free diets, etc (58–60); according to a meta-analysis, continuing for 6 months helped people on a gluten-free diet improve thyroid function, reduce thyroid inflammation levels, and lower antibody levels (61).

## Summary and future perspectives

The pathogenesis of HT involves a complex interaction of genetic susceptibility, immune regulation, and environmental factors. In recent years, with the continuous development of science and technology, our understanding of the disease has deepened, and the diagnosis and treatment of the disease are no longer limited. However, this is far from enough, and we need to further explore, for example, new biomarkers that can assess the efficacy of treatment and the mechanisms of action of different therapeutic approaches. Due to the TPOAb/TgAb assay limitations, it is recommended to develop a composite marker that combines immunocytology and imaging. And predict the value of this marker for the regression of subclinical hypothyroidism should be verified by a prospective cohort. For patients in the subclinical stage, based on are recommended immunocytology randomized controlled trails to immune markers assess selenium supplementation whether immunomodulatory regimens such as can delay disease progression by regulating the Treg/Th17 balance. For the clinical efficacy of traditional Chinese medicine, such as Xiaoyao-san, it is recommended to verify whether the mechanism of action is carried out by regulating the balance of Treg/Th17 through animal experiments. And to clarify the mechanism of intestinal flora in lowering the level of thyroid antibodies. Through the above pathways, it may be possible to promote the transformation of HT diagnosis and treatment from passive replacement to active intervention, and from vague diagnosis to precision medicine. This still requires the joint participation and unremitting efforts of medical workers and researchers.
